# Do avian blood parasites influence hypoxia physiology in a high elevation environment?

**DOI:** 10.1186/s12898-018-0171-2

**Published:** 2018-05-14

**Authors:** Farah Ishtiaq, Sahas Barve

**Affiliations:** 1 Centre for Ecological Sciences, Indian Institute of Science, Bangalore, 560012 India; 2 Department of Biological Sciences, Old Dominion University, Norfolk, VA 23529 USA

**Keywords:** Anaemia, *Haemoproteus*, Hypoxia, Haemoglobin, Haematocrit, Infection status, *Plasmodium*, *Leucocytozoon*, Western Himalaya

## Abstract

**Background:**

Montane birds which engage in elevational movements have evolved to cope with fluctuations in environmental hypoxia, through changes in physiological parameters associated with blood oxygen-carrying capacity such as haemoglobin concentration (Hb) and haematocrit (Hct). In particular, elevational migrants which winter at low elevations, encounter varying intensities of avian haemosporidian parasites as they traverse heterogeneous environments. Whilst high intensity parasite infections lead to anaemia, one can expect that the ability to cope with haemosporidian infections should be a key trait for elevational migrants that must be balanced against reducing the oxygen-carrying capacity of blood in response to high elevation. In this study, we explored the links between environmental hypoxia, migration, and disease ecology by examining natural variation in infections status and intensity of avian haemoporidians across a suite of Himalayan birds with different migratory strategies while controlling for host phylogeny.

**Results:**

We found predictably large variation in haemoglobin levels across the elevational gradient and this pattern was strongly influenced by season and whether birds are elevational migrants. The overall malaria infection intensity declined with elevation whereas Hb and Hct decreased with increase in parasite intensity, suggesting an important role of malaria parasites on hypoxia stressed birds in high elevation environments.

**Conclusions:**

Our results provide a key insight into how physiological measures and sub-clinical infections might affect dynamics of high-elevation bird populations. We suggest a potential impact of avian elevational migration on disease dynamics and exposure to high intensity infections with disease spread in the face of climate change, which will exacerbate hypoxic stress and negative effects of chronic avian malaria infection on survival and reproductive success in wild birds. Future work on chronic parasite infections must consider parasite intensity, rather than relying on infection status alone.

**Electronic supplementary material:**

The online version of this article (10.1186/s12898-018-0171-2) contains supplementary material, which is available to authorized users.

## Background

Environmental hypoxia—the decreased partial pressure of oxygen—and cold temperature are key drivers in the evolution of high elevation adaptations in montane organisms [1]. In particular, birds that undertake elevational migrations are exposed to considerable seasonal fluctuations in hypoxia levels and temperature across the year. Therefore elevational migrants must evolve physiological strategies to regulate haemoglobin (hereafter Hb) concentration, an important measure of blood oxygen-carrying capacity [2], as they move between elevations. High intensity infections with avian ectoparasites and haemoparasites have been shown to lower the oxygen-carrying capacity of their hosts by reducing Hb levels through direct consumption or destruction of red blood cells, often leading to anaemia [3]. Thus the capacity to regulate Hb concentration and cope with hypoxia can be significantly compromised by haemoparasite infection in elevational migrants which are at increased risk of infection due to the following several factors. Elevational migrants tend to encounter more parasites as they are exposed to high prevalence areas at low elevations with optimal climatic conditions for parasite transmission (e.g. [4]), have increased physiological demands incurred due to changes in immune conditions during migration ([5] but see [6]), have a relapse of chronic infections prior to or during the journey [7], and have increased risk of exposure due to host aggregation during migration or at the wintering sites [8]. Given the physiological strategies and life-history characteristics, high intensity parasite infections could be especially costly in hypoxic environments for montane birds. Recent evidence suggests that Hb is a more reliable indicator of host response to parasitism than lowered haematocrit (Hct, total red blood cell volume), due to the commonly observed occurrence of erythropoiesis in parasitized hosts [3]. In birds, regenerative or haemolytic anaemia leads to increased erythropoiesis that is rapid production of immature red blood cells stimulated by hypoxia and yet showing normal Hct, despite a substantial parasite load (intensity). To date, only a few studies have compared association between Hb levels and ectoparasite load [9–11] and haemoparasites [12], and these have only been in juvenile altricial birds [12].

Avian haemosporidians (Apicomplexa: Haemosporida; *Plasmodium* and other related genera *Haemoproteus* spp. and *Leucocytozoon* spp.; henceforth referred to as ‘avian malaria’) are vector-borne blood parasites that place significant selective pressure on their host populations by reducing reproductive success [13], immune response [14, 15], and chronic-infection driven fitness [16]. Increased parasite intensities can have negative effects on bird populations by reducing growth [17] and causing higher mortality and/or lower birth rates [18].

The temporal and spatial dynamics, and epidemiology of avian malaria are strongly governed by ecological (season, habitat quality, elevation) [19, 20], demographical (host and vector density) [21], and environmental factors (temperature, precipitation) [22]. Seasonal variation in ambient temperature, influences host condition through an increase in energy demands [23], leading to stress, and thereby increasing susceptibility to infection [24]. In addition, host and vector abundance govern the frequency-dependent transmission of the pathogens [22, 25–28]. Cosgrove et al. [29] showed a marked seasonality in *Plasmodium* transmission in temperate environments, which appears to result in zero prevalence estimates in winters. This is in stark contrast to the tropics where there is a lack of seasonal variation in *Plasmodium* spp. prevalence in birds despite a strong correlation between mosquito abundance and temperature [28]. Given these seasonal effects, the role of avian haemosporidians on their host has mainly been examined in the breeding season where the spring–summer emergence of parasitemia in avian hosts coincides with a high allocation of energy in reproduction, and high levels of corticosterone in the blood. However, the impact of parasite infection in the non-breeding season has largely remained neglected but see [30]. Furthermore, climate change is likely to facilitate expanding geographic range of *Plasmodium* species with an increase in the transmission window in habitats that are currently too cool to sustain vector populations and parasite development.

The western Himalayan bird community provides a unique opportunity to understand the interplay between hypoxia and blood parasite intensity. In this montane system, birds exhibit two migration strategies; species are either year-round high elevation residents (sedentary) or seasonal elevational migrants. Elevational migrants winter at low elevations or in the plains (≤ 1500 m above sea level; a.s.l.) and move to breeding grounds at higher elevations (2600–4000 m a.s.l. or even higher) during the summer season [31]. In the wintering grounds, resident birds may act as reservoirs for blood parasites, increasing the risk of infection for migrants. Elevational migrants thus, encounter a more diverse fauna of parasites compared with sedentary species. Barve et al. [32] showed that high elevation residents regulate Hb by having high mean cell haemoglobin concentration (MCHC), while elevational migrants adjust their Hb through fluctuation in Hct as they move between high and low elevations. The degree to which elevation and blood parasites influence Hb levels across elevational migrants and resident birds has remained unexplored and can have important implications for the ecology of parasites and montane birds. Given that thermal conditions and suitable vectors are present to transmit and maintain the infection, such migrants can form an effective bridge for parasites between the wintering and breeding grounds. Hence, this increases the risk of infection to naïve resident birds at high elevations which might not have evolved to cope with decreased Hb levels as a result of parasite infection. Additionally, elevational migrants are exposed to hypoxia for a relatively short period compared to their high elevation resident counterparts. Therefore, an examination of Hb concentration and haemosporidians infections in resident and migrant species can reveal whether physiological stress and haemolytic anaemia might be more detrimental to high elevation residents, with more haemoglobin lost with the haemolysis of each red blood cell, exacerbating physiological stress across a large elevational gradient.

In this study we examined the infection status and infection intensity of three parasite genera *Plasmodium*, *Haemoproteus* and *Leucocytozoon* in 18 Himalayan avian species which differ in their migration strategies, and their erythropoietic response to hypoxia. We predicted that: (i) seasonal variation in probability of infection and infection intensity will be higher in elevational migrants than their resident counterparts; (ii) infection status and infection intensity will show a negative relationship with Hct and Hb levels; and (iii) high infection intensity and low Hb levels will be associated with elevational migrants as they are likely to experience more chronic hypoxia in the breeding season than high elevation residents.

## Methods

### Study site, avian blood sampling and physiological parameters

**Fig. 1:**
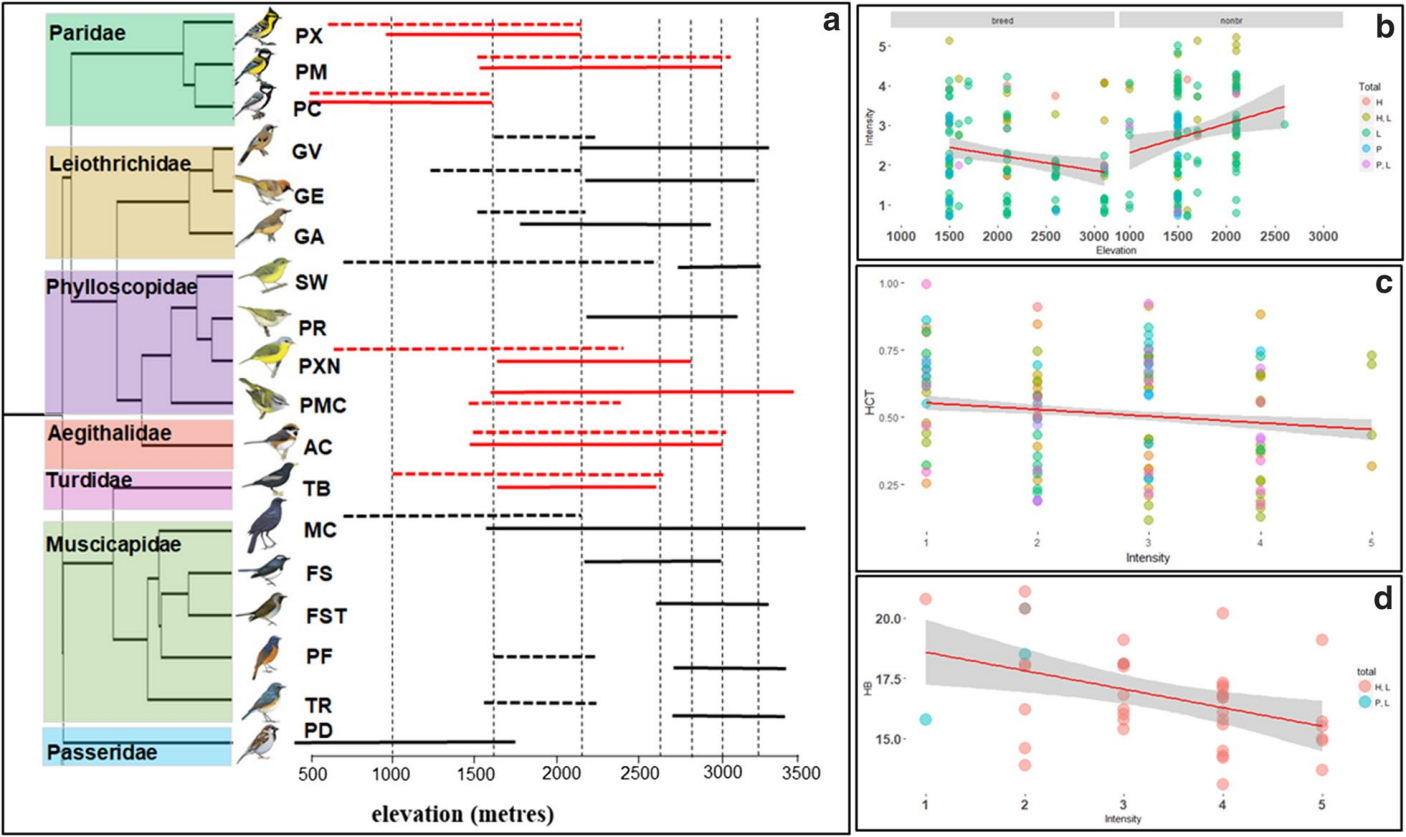
**a** Phylogenetic relationships among bird species used in the analysis following [46]. Elevational distributions are from Dixit et al. [31] and Rasmussen and Anderton [41]. Dotted vertical lines represent sampling locations. Horizontal solid lines represent species breeding elevational distribution and dashed lines represent species winter distribution (elevation in metres) of resident (red) and elevational migrant (black) species. Coloured boxes on the left margin denote taxonomic families used in the analysis. Species codes (from top): PX (*Parus xanthogenys*, n = 9), PM (*Parus monticolus*, n = 115), PC (*Parus cinereus*, n = 24), GV (*Garrulax variegatum*, n = 29), GE (*Garrulax erythrocephalus*, n = 64), GA (*Garrulax albogularis*, n = 30), SW (*Seicercus whistleri*, n = 10), PR (*Phylloscopus reguloides*, n = 27), PXN (*Phylloscopus xanthoschistos*, n = 83), PMC (*Phylloscopus maculippenis*, n = 11), AC (*Aegithalos concinnus*, n = 41), TB (*Turdus boulboul*, n = 8), MC (*Myophonus caeruleus*, n = 43), FS (*Ficedula superciliaris*, n = 6), FST (*Ficedula strophiata *= n=14), PF (*Phoenicurus frontalis*, n = 15), TR (*Tarsiger rufilatus*, n = 24), PD (*Passer domesticus*, n = 20). Bird illustrations were reproduced from Handbook of the Birds of the World Alive [78]. **b** Total parasite intensity decrease with increase in elevation (m). Parasite intensity increases with mid-elevation in non-breeding season. Intensity: 1 = submicrocopic; 2 = low; 3 = medium; 4 = high; 5 = very high; H = *Haemoproteus*, L = *Leucocytozoon*, P = *Plasmodium*, H, L = *Haemoproteus* and *Leucocytozoon* co-infections, P, L = *Plasmodium* and *Leucocytozoon* co-infections. **c** Hct is negatively correlated with total parasite intensity. **d** Hb is negatively correlated with parasite intensity. H, L = *Haemoproteus* and *Leucocytozoon* co-infections, P, L = *Plasmodium* and *Leucocytozoon* co-infections

The bird blood sampling was conducted in the non-breeding season (January–March) and breeding season (April–May) in Kedarnath Wildlife Division, Uttarakhand, India. We selected 18 species of passerine birds based on their abundance and diversity across seven elevations: 1000, 1500 (low), 2100 (mid), 2650, 2800, 3000 and 3200 m (high) a.s.l. as mentioned in Barve et al. [32] (Fig. 1a, Additional file 1: Table S1).

At each sampling site, 6–12 mist nets were set up in high bird activity locations. Each bird caught was identified and ringed with a numbered metal ring. Bird blood from the sub-brachial wing vein (never exceeding 1% of the individual’s body weight) was collected in SET Buffer (20–40 μL in 500 μL buffer 0.15 M NaCl, 0.05 M Tris, M EDTA, pH 8.0) or FTA cards (Whatman) for molecular analyses. Captured individuals were released at the site immediately after processing. In addition, for each blood sample, the concentration of Hb was determined using a portable HemoCue Hb 201+ photometer (HemoCue Hb, Ängelholm, Sweden). In this study we measured Hb for 18 bird species and obtained 286 samples in the breeding season and 287 in the non-breeding season (Additional file 2: Table S2 and Fig. S1 for summary of bird species with infection status and Hb values).

Whenever a sufficient sample (20–30 μL) was available, we also measured Hct (n = 245; Additional file 2: Table S2) for the same samples, using a Zipocrit Portable Centrifuge (LW Scientific Inc., Lawrenceville, GA, USA) with a spin time of 5 min. Both Hb and Hct measurements were highly repeatable within this study [32].

In addition, for the 2014–2015 bird sampling in the breeding season (April–May), thin blood smears were prepared on glass slides to identify whether infections exhibited parasite gametocytes, which would indicate that elevational migrants act as ‘true’ hosts for that particular parasite strain [33]. The smears were air-dried, fixed in 100% methanol and stained with Giemsa.

### Infection status and intensity estimation

DNA extractions were conducted using the phenol chloroform extraction method [34]. The presence of *Plasmodium*, *Haemoproteus* and *Leucocytozoon* was assessed using parasite-specific primers designed to amplify partial fragments the cytochrome *b* (cyt-*b*) gene using nested PCR protocol [35]. Each plate run included a parasite positive control and also a negative control (water instead of template DNA) to examine for any potential contamination. We screened all parasite negative samples for bird DNA (for cyt-*b* gene) following Dumbacher et al. [36]. The resulting PCR products were then sequenced in both directions. Sequences were assembled, aligned and edited using SEQUENCHER version 5.2 (Genecodes Corp., Ann Arbor, MI, USA). We then identified sequences to genus using their closest sequence matches in the GenBank or MalAvi databases [37]. For each positive PCR product (without blood smear in non-breeding season; January–March), parasite intensities were calculated as relative quantification values (RQ) as follows. We performed two quantitative PCRs (qPCR): one targeting the 18sRNA gene of avian haemosporidians following Ishtiaq et al. [38], and the other targeting the 18sRNA gene of the bird (as described in [39]). RQ can be interpreted as the fold-amount of the target gene (parasite 18s rDNA) with respect to the amount of the reference gene (Bird18s rDNA) and was calculated as 2^−(Ct18s parasite−Ct18s Bird)^ where Ct represents the number of PCR cycles at which fluorescence was first detected as statistically significant above the baseline, which is inversely correlated with the initial amount of DNA in a sample. Each DNA sample was run in duplicate and the average values were used for further analysis. For samples run on different plates we used one reference sample common to all plates. We ran all samples, including the inter run calibrator (IRC), in duplicate. Runs were validated only if the non-template control (NTC) and the negative control did not exhibit fluorescence curves that crossed the threshold line, and the positive control gave a fluorescence curve that crossed the threshold line within 38 cycles (Ct ≤ 38). Following each run, we examined the results to ensure that replicates had similar Ct values and melting peaks. We validated intensity data against microscopy by including positive samples which had corresponding blood smear to calculate intensity. These results showed a good correspondence between parasitemia estimated by qPCR and microscopy [38] which allowed us to compare intensity results using qPCR and traditional microscopy techniques. Similar results have been reported in other studies (e.g. [16]). For convenience, RQ values were log transformed into intensity categories so it can be combined with the data with intensities scored on smears (see below). For example, assuming that in one microscopic field is 300–400 erythrocytes, then: ++++ should be more than approx. 3% (in percentage; 3 of 10 erythrocytes are infected); +++ should be about 0.3–1.3%, ++ should be about 0.03–0.2%, + should be about 0.003–0.02%. So, qPCR values (host/parasite ratio) of 1.796519336 were considered as +++; 0.011073119 + 0.064422307 ++. This way we generated intensity data for all samples ranging from submicroscopic, low (+), medium (++), high (+++) to very high (++++) intensity.

### Light microscopy

Blood smears were examined for the presence of infected blood cells and gametocytes following Godfrey et al. [40]. Briefly, all slides were first examined at low magnification (500×) for approximately 100 fields and then at least 100 fields were studied at high magnification (1000×) using an oil immersion lens (taking into account the 10× contribution from the ocular combined with objectives of the magnification reported). The intensity of infection was recorded as per the following criteria: low (+); 1–10 parasites per 100 thin film fields, medium (++); 11–100 parasites per 100 thin film fields, high (+++); 1–10 parasites in one thin film field and very high (++++); more than 10 parasites in one thin film field.

### Modelling disease risk in Hb compromised species

For comparison of infection status across three parasite genera, we carried out four distinct analyses. We classified each sampled species as either an elevational migrant (EM status = 1) where almost all individuals migrate to significantly lower elevations or as sedentary (EM status = 0) were species that are consistently found in their breeding range in the winter in our study site. Information on elevational movement and distribution was obtained from a field survey [31] in our study site and Rasmussen and Anderton [41]. The first analysis consisted of generalized linear mixed models (GLMMs, function glmer in lme4; Bates et al. [42]) to assess whether individual infection status with *Plasmodium*, *Haemoproteus* and *Leucocytozoon* was influenced by elevation, bird migration strategy and season, as fixed effects, and bird taxonomy (species nested in genus and genus nested in family) as a random effect. All models were specified with a binomial error distribution and logit link function. The significance of fixed effects was evaluated with Wald’s χ^2^-tests [43].

In the second analysis, we used a linear mixed effect models to assess differences in infection intensity of the three parasite genera (*Plasmodium*, *Haemoproteu*s and *Leucocytozoon*) with elevation, season and migration status as fixed effects, and bird species as a random effect. For the intensity data, we used only positive samples with microscopy and qPCR methods. We further analysed the relationship between infection status and parasite intensity with Hct sampled across species. Owing to limited data, Hct was not analysed for migration strategies and season. Finally, we sought to tease apart the effects of migration status, parasite intensity, elevation, and season, on Hb levels. In order to investigate factors relating to Hb (at a variety of complexities/scales), we constructed predictive regression models using the linear mixed-effects kinship model fit by maximum likelihood (lmekin) within the “coxme” package [44]. For each model, we controlled for phylogenetic effects by including a phylogenetic correlation matrix as a random effect using the “ape” package [45]. The phylogenetic correlation matrix was derived from a phylogeny constructed from information by Jetz et al. [46]. All data was checked for normality and corrected for over-dispersion as required.

Prior to running predictive models, correlations between predictor variables were assessed to test for collinearity using correlation matrices and simple linear models generated for each pairwise combination of predictors. There was a significant positive relationship between Hb and elevation in both elevational migrants (R^2^ = 0.24, *P* < 0.001) and residents (R^2^ = 0.16, *P* < 0.001; [32]). We used intensity as an interactive term in all models since infection intensity can vary with time, seasons, age and sex [20, 47] and showed a negative association with Hb levels (see below). All other pairwise comparisons yielded absolute correlation values and variance explained by linear models (adjusted R^2^) of < 0.5, indicating no issues of collinearity, so all predictors were retained.

Using data for 15 species in summer and nine species in winter (18 species in total), we fitted 18 linear mixed models separately for *Leucocytozoon*, *Plasmodium*, *Haemoproteus*, mixed infections and total infected birds. We used AIC-based multi-model inference to identify well-supported statistical models that describe the relationships between Hb and biological parameters relevant to elevational distribution, such as (i) infection intensity, (ii) elevation (low, mid, high), (iii) EM status (1 = elevational migrants, 0 = resident species), (iv) season (breeding = 1, nonbreeding = 0), as fixed effects. In all cases, bird species was included as a random effect. The models were written based on variables that were ecologically relevant to hypoxia physiology (Additional file 3: Table S3) and model selection was done using the MuMIn package [48].

We used the Akaike Information Criteria (AIC; see [49]), to select the best-fit model. We generated a set of 18 candidate models (including global model and single predictor models) which were ranked by AICc (Additional file 3: Table S3). Models with a difference (ΔAICc) of ≤ 2 are as parsimonious as the best-fit model (lowest AICc), and is considered strong evidence that the quality of the candidate models differ from one another [49]. Relative importance of the traits was assessed and model-averaged estimates were derived to account for model uncertainty [50]. We used a model-averaging approach to check the validity of the top-ranking model in each case, only including models with ΔAICc < 2 [49]. The relative importance (RI) of each parameter after model-averaging, was calculated by summing *wi* across all models in which the parameter was present. Analyses were conducted in R v. 3.0.1 [51].

## Results

Of the 573 birds sampled, 44.8% (95% CI 40.7–48.9%) were positive for avian haemosporidians infections. *Leucocytozoon* spp. showed significantly higher infection prevalence (39.7%; 95% CI 35.9–43.9%) than *Haemoproteus* spp. (10.2%; 95% CI 7.8–12.7%) and *Plasmodium* spp. (3.1%; 95% CI 1.7–4.5%) (Wald’s χ^2^ = 232.88, *df* = 2, P < 0.0001). Only 7.3% (95% CI 5.1–9.4%) were co-infections.

### Seasonal variation in the probability of infection and parasite intensity

In the first analysis, across the three parasite genera, there was no significant difference in the infection status with elevation, season and migration status of species (Table 1). However, the probability of *Plasmodium* infection was high for low elevations, whereas *Leucocytozoon* infection risk increased with elevation, albeit as submicroscopic infections. In the second analysis the infection intensities of *Leucocytozoon* (Wald’s χ^2^ = 15.70.12, *df* = 1, *P *< 0.0001) was higher in non-breeding than in the breeding season, but with no effect of bird migration status (Table 2). Total parasite intensity showed a significant decrease with elevation (Wald’s χ^2^ = 7.12, *df* = 2, *P *< 0.01; Fig. 1b) in the breeding season. Birds had a higher infection intensity in the non-breeding season across mid-elevation (Wald’s χ^2^ = 6.06, *df* = 2, *P *< 0.04; Table 2).

**Table 1:** GLMM (generalized linear mixed model with binomial error distribution and logit link function) to test the influence of elevation, migratory status (elevational migrant versus resident) and season (breeding versus non-breeding) as fixed effects on the probability of infection with *Plasmodium* spp., *Haemoproteus* spp., *Leucocytozoon* spp., total infections and mixed infections after controlling for host taxonomy as random effect

	β	SE	z value	P
Infection risk with parasite				
(A) Total infection				
(Intercept)	− 0.399	0.46	− 0.85	0.39
Season (non-breeding)	0.015	0.23	0.06	0.94
Status (resident)	− 0.006	0.44	− 0.01	0.98
Elevation_medium	− 0.001	0.26	− 0.006	0.99
Elevation_high	0.65	0.34	1.88	0.05
(B) *Leucocytozoon*				
(Intercept)	− 0.76	0.48	− 1.58	0.11
Season (non-breeding)	0.09	0.23	0.39	0.69
Status (resident)	− 0.17	0.48	− 0.36	0.71
Elevation_medium	0.10	0.28	0.38	0.69
Elevation_high	0.69	0.37	1.87	0.06
(C) *Haemoproteus*				
(Intercept)	− 3.35	0.92	− 3.63	*0.0001*
Season (non-breeding)	0.29	0.36	0.78	0.43
Status (resident)	− 1.55	1.15	− 1.34	0.17
Elevation_medium	0.54	0.37	1.44	0.14
Elevation_high	0.78	0.50	1.57	0.11
(D) *Plasmodium*				
(Intercept)	− 3.38	0.68	− 4.96	*0.0001*
Season (non-breeding)	− 0.30	0.61	− 0.49	0.62
Status (resident)	0.56	0.57	0.99	0.32
Elevation_medium	− 1.66	1.06	− 1.57	0.11
Elevation_high	− 0.53	0.86	− 0.62	0.53
(E) Mixed infections				
(Intercept)	− 3.36	0.78	− 4.27	*0.0001*
Season (non-breeding)	0.35	0.41	0.83	0.40
Status (resident)	− 1.64	0.99	− 1.65	0.09
Elevation_medium	0.27	0.43	0.64	0.52
Elevation_high	0.45	0.58	0.77	0.44

Significant values are in italics

**Table 2:** Linear mixed model to test the influence of elevation, migratory status (elevational migrant versus resident) and season (breeding versus non-breeding; nonbr) as fixed effects on avian haemosporidian intensity after controlling for host taxonomy

	β	SE	*df*	t value	P
Total infection intensity (n = 257, 18 species)					
(Intercept)	2.60	0.21	28.15	12.15	*0.0001*
Elevation_medium	− 0.44	0.29	242.65	− 1.48	0.14
Elevation_high	− 0.76	0.26	205.26	− 2.89	*0.001*
Season (non-breeding)	0.12	0.19	237.21	0.62	0.53
Status (resident)	− 0.45	0.24	12.95	− 1.84	0.08
elev_med:season_nonbr	0.78	0.33	247.76	2.37	*0.01*
elev_high:season_nonbr	1.00	1.09	243.56	0.91	0.35
elev_med:status_resident	0.34	0.38	214.28	0.90	0.36
elev_high:status_resident	0.39	0.44	243.43	0.88	0.37
*Leucocytozoon* intensity (n = 229, 17 species)					
(Intercept)	2.57	0.24	22.35	10.49	*0.0001*
Elevation_medium	− 0.44	0.31	219.63	− 1.40	0.16
Elevation_high	− 0.79	0.28	197.27	− 2.77	*0.001*
Season (non-breeding)	0.10	0.20	217.56	0.50	0.61
Status (resident)	− 0.39	0.29	11.27	− 1.34	0.20
elev_med:season_nonbr	0.76	0.35	219.76	2.18	*0.01*
elev_high:season_nonbr	1.03	1.09	212.50	0.94	0.34
elev_med:status_resident	0.11	0.41	204.50	0.27	0.78
elev_high:status_resident	0.11	0.50	218.69	0.22	0.82
*Haemoproteus* intensity (n = 59, 9 species)					
(Intercept)	1.82	0.53	22.48	3.42	*0.001*
Season (non-breeding)	0.21	0.32	53.37	0.66	0.50
Status (resident)	− 0.93	0.76	14.68	− 1.22	0.23
Elevation_medium	0.63	0.33	55.36	1.88	0.06
Elevation_high	− 0.06	0.42	53.15	− 0.15	0.87
*Plasmodium* intensity (n = 18, 9 species)					
(Intercept)	2.50	0.68	5.80	3.68	*0.01*
Season (non-breeding)	0.38	0.51	12.98	0.75	0.46
Status (resident)	− 0.78	0.80	6.20	− 0.97	0.36
Elevation_medium	1.11	1.25	10.30	0.88	0.39
Elevation_high	− 1.00	0.96	5.80	− 1.03	0.34
Mixed infection intensity (n = 42, 8 species)					
(Intercept)	3.07	0.37	37	8.14	*0.0001*
Season (non-breeding)	0.42	0.39	37	1.07	0.28
Status (resident)	− 1.00	0.57	37	− 1.74	0.08
Elevation_medium	0.43	0.37	37	1.16	0.25
Elevation_high	− 0.21	0.55	37	− 0.39	0.69

Significant values are in italics

### Effect of parasite intensity on Hct and Hb

**Fig. 2:**
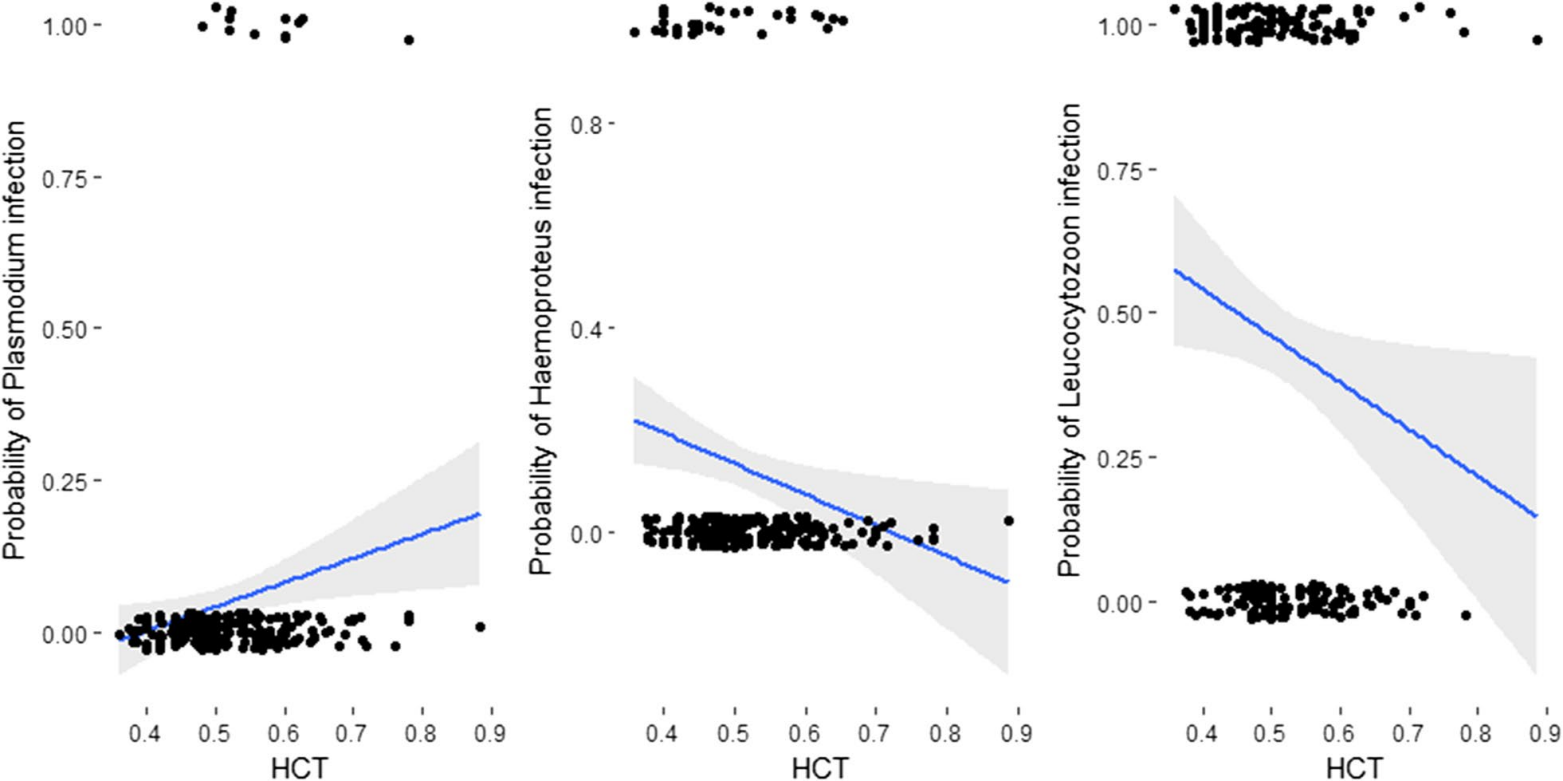
Relationship between haematocrit (Hct) and the probability of infection with avian haemosporidians

In the third analysis, there was a significant negative relationship between Hct and overall avian malaria intensity (*b *= − 0.01, *t *= − 2.45, *P *< 0.02; Fig. 1c). The probability of infection with *Plasmodium* spp. showed significant increases with Hct (Wald’s χ^2^ = 4.54, *df* = 1, *P *< 0.03; Fig. 2). However, neither *Haemoproteus* spp. (Wald’s χ^2^ = 1.69, *df* = 1, *P *= 0.19) nor *Leucocytozoon* spp. (Wald’s χ^2^ = 0.11, *df* = 1, *P *= 0.73) showed significant variation in infection status with Hct. The total parasite intensity as a single predictor showed a negative relationship with Hb (*b *= − 0.22, *t *= − 2.68, *P *< 0.001).

In the final analysis, for total parasite intensity, model-averaged estimates derived from 90% model set agreed with the best-approximating model with high elevation, elevational migrant and interaction of breeding season and intensity. High Hb levels were influenced by high elevation, elevational migrant and breeding season. Although, for *Leucocytozoon* infected birds, the model-averaged estimates indicated that high elevation influenced the high Hb levels, however, parasite intensity appeared to have negative effect on Hb. For *Haemoproteus* infected birds, none of the parameter showed any influence on Hb. However, for *Plasmodium* infected birds, Hb was positively influenced by elevational migrants. Mixed infections showed the negative influence of parasite intensity on Hb levels as the top model (*b *= − 0.54, *t *= − 2.78, *P *< 0.001; Fig. 1d; Table 3).

**Table 3:** Model-averaged predictive models for effect of each parameter on Hb levels: model-averaged fixed effects parameter estimates for: (a) total infections, (b) *Leucocytozoon*, (c) *Haemoproteus*, (d) *Plasmodium*, and (e) mixed infections with relative importance (RI) of each parameter

Parameter	Estimate (β)	SE	Z-value	*P* value	Confidence interval	RI
(a) Total infections (n = 257)						
(Intercept)	17.20	0.95	18.07	*<* *0.0001*	(15.34, 19.07)	NA
Intensity	− 0.22	0.12	1.75	0.07	(− 0.47, 0.02)	1.00
Breed (1 = breeding season)	− 0.70	0.49	1.42	0.15	(− 1.66, 0.26)	1.00
Elevation_medium	0.35	0.23	1.53	0.12	(− 0.09, 0.80)	1.00
Elevation_high	0.96	0.32	3.00	*0.001*	(0.33, 1.59)	1.00
EM (1 = elevational migrant)	1.52	0.77	1.96	*0.04*	(0.007, 3.05)	1.00
Breed: intensity	0.36	0.17	2.11	*0.03*	(0.02, 0.69)	1.00
EM: intensity	− 0.09	0.17	0.51	0.60	(− 0.43, 0.25)	0.28
(b) *Leucocytozoon* (L) (n = 229)						
(Intercept)	18.71	1.05	17.70	*<* *0.0001*	(16.64, 20.78)	NA
*L*_intensity	− 0.25	0.11	2.17	*0.02*	(− 0.48, − 0.02)	0.81
Breed (1 = breeding season)	− 0.38	0.60	0.64	0.52	(− 1.58, 0.80)	0.81
Elevation_medium	0.31	0.24	1.26	0.20	(− 1.72, 0.79)	1.00
Elevation_high	1.07	0.35	3.05	*0.001*	(0.38, 1.76)	1.00
EM (1 = elevational migrant)	− 1.02	0.65	1.56	0.11	(− 2.31, 0.26)	0.81
Breed: *L*_intensity	0.32	0.18	1.76	0.07	(− 0.03, 0.69)	0.49
(c) *Haemoproteus* (H) (n = 59)						
(Intercept)	17.42	1.98	8.72	*<* *0.0001*	(13.52, 21.31)	NA
*H*_intensity	− 0.03	0.47	0.08	0.93	(− 0.96, 0.88)	0.73
EM (1 = elevational migrant)	3.36	1.88	1.78	0.07	(− 0.32, 7.05)	0.50
EM: *H*_intensity	− 0.83	0.43	1.90	0.05	(− 1.68, 0.02)	0.38
Breed (1 = breeding season)	− 0.53	1.37	0.39	0.69	(− 3.22, 2.15)	0.36
Breed: *H*_intensity	0.53	0.36	1.46	0.14	(− 0.17, 1.24)	0.22
(d) *Plasmodium* (P) (n = 18)						
(Intercept)	17.33	0.48	35.51	*<* *0.0001*	(16.37, 18.28)	NA
EM (1 = elevational migrant)	2.47	0.60	4.10	*0.0001*	(1.29, 3.65)	0.73
Breed (1 = breeding season)	0.98	0.52	1.88	0.05	(− 0.03, 2.00)	0.27
(e) Mixed infections^a^ (n = 42)						
Intensity	− 0.54	0.19	− 2.78	*0.005*		

^a^Parameter estimate based on the best model

## Discussion

This is the first study which links interspecific variation in migratory strategies, variation in erythropoietic responses to hypoxia and parasite infection and intensity across an elevational gradient. Our current understanding of the seasonality and epidemiology of the avian haemosporidian community has been largely derived from studies conducted in the breeding season in temperate regions, when parasite infections peak in spring and summer with an increase in vector activity [52, 53]. Our study on western Himalayan birds shows that parasites persist in winters and infections are higher in intensity in the non-breeding season. We also demonstrate that high parasite intensity has a negative influence on both Hb and Hct suggesting a role of haemosporidian infections in impacting hypoxic stress in high elevation Himalayan birds.

### Seasonal variation in parasite intensity and infection status

The presence of parasitemia throughout our study period (January–May; 2014–2015) suggests various underlying mechanisms; (i) the continued presence of all three parasite genera in medium to high intensity during the non-breeding season, highlighting the environmental stress in winters which likely induces a relapse of the previous year’s infections [52, 54]; (ii) scarcity of food in highly seasonal habitats like the Himalayas (e.g. [55]), which directly affects body condition [56, 57], and is often reflected in high levels of infection intensity in winters. Migrant birds generally have poorer condition in winters compared to summer [30] and body condition can deteriorate with high intensity infections [18]. In addition, Hb declines with haemosporidian infections [3], and a lower Hb with high intensity infections mainly driven by *Leucocytozoon* spp. in the non-breeding season, would reflect a seasonal decline in trophic conditions. High intensity infections in elevational migrants in winter is thus an important finding, which has important implications for seasonal disease dynamics with the potential for carryover effects to the breeding season. Many elevational migrants sampled at high elevations during the breeding season were infected with submicroscopic infections.

Dunn et al. [30] showed high incidence of *Haemoproteus* spp. in the non-breeding season, however, at low intensity. In our study, total parasite intensity was high in the non-breeding season. *Plasmodium* spp. intensity showed no variation across seasons which is likely to be chronic stages of infections, whereas *Leucocytozoon* spp. showed high intensity in the non-breeding season regardless of bird migration status, which can be classified either as chronic or relapses. These findings have important implications for over-winter ecology of *Leucocytozoon* spp. in elevational migrants. Nonetheless, the total parasite intensity showed a significant decrease with elevation despite no change in parasite infection status with elevation. Temperature is a key driver of haematophagous arthropod vectors and parasite life-history traits that combine to determine transmission intensity. Even though the probability of infection with three parasite genera at these sites is variable, the sampling sites were selected primarily to illustrate the contrasting thermal environments that can exist across relatively small spatial scales within a region. Our sampling sites vary in elevation (Fig. 1a) and have marked differences in mean temperatures and diurnal temperature ranges, and thus have divergent effects on parasite development and persistence at a population level [58]. The daily temperatures in the low elevation sites during the non-breeding season were warmer (10–25 °C) than mid to high elevation sites (4–10 °C; FI unpub. data) which suggests that low elevations have optimal conditions and lack any thermal constraints for the parasites and this probably allows the parasites to be present in the blood stream during non-breeding season. This would imply an ongoing transmission at the low elevation during winter as long as potential vector species are present, whereas at colder higher elevation sites, parasites withdraw from the blood stream until the ideal conditions appear.

### Effect of parasite intensity on Hct and Hb

In western Himalayan birds, high Hb levels were strongly associated high elevation, and the breeding season where elevational migrants are likely to experience chronic hypoxia (potentially compounded by increased energetic demands) [32]. In our linear mixed model analyses to examine factors influencing Hb levels in parasite-specific infected bird models, parasite intensity was selected as an important predictor in mixed infection birds, however, parasite intensity showed negative significant effect in *Leucocytozoon* infected birds with confidence intervals for the parameter estimates included zero, so there was little evidence that *Leucocytozoon* parasite had any effect on Hb levels. These results are consistent with other studies that report negative effects of parasite intensity on haemoglobin concentration in birds [3, 18, 59]; whereas some studies report no effect of parasite intensity on Hb levels [60]. Based on a recent experimental study, even low intensity chronic infection influences physiology and fitness of hosts [61]. Therefore, measurement of parasite intensity is important rather than relying only on infection status in evaluating the role of infections on host fitness and physiology.

Whilst the prevalence estimates of haemosporidians are snapshots in time and space [62, 63], our findings are important from physiological as well as ecological perspectives. Previous studies have shown that there is a correlation between parasite intensity and the severity of human malarial infections-high parasite intensity is correlated with high mortality rates [64]. It is quite possible that high intensity blood parasite infections are pathogenic to elevational migrants and lead to excessive haemolysis of parasitized erythrocytes in malaria infection, and may lead to anaemia (e.g. [65, 66]). This is further supported by the low frequency of very high intensity parasite infections in high elevation sites. Many elevational migrants were detected with submicroscopic infection (detected by PCR but not by microscopy and this implies absence of infective parasite stage—gametocytes) in high elevation environments in peak breeding season despite being found in medium or high intensity at low elevations. These findings are in stark contrast with other studies in temperate regions where the majority of the birds with latent infections (birds infected during the prior breeding season) return to the breeding grounds and experience an increase in parasite blood stages—relapse [54, 67]. Parasite intensity in *Plasmodium falciparum* often shows a clear trend of decreasing with decreasing transmission intensity [68] and our finding implies that elevation can be a proxy for transmission intensity in avian haemosporidians, highlighting that studies should not only rely on prevalence based models as intensity drives the transmission intensity in high elevation sites and the combination of molecular tools and microscopy is critical in assessing parasite load. While we quantified relative intensity across Himalayan birds, one of the limitations of using mix intensity data—by converting qPCR values into intensity categories as scored on smears—meant loss of quantitative information which would have provided better resolution. Our estimates could be compromised for genus level intensity in few co-infected birds as qPCR primers amplify all three parasite genera successfully [39]. Nonetheless, our parasite intensity estimates from qPCR and microscopy techniques were comparable as we screened all samples using nested PCR protocols [38, 39].

Similarly, Hct, an important predictor of blood oxygen transport in elevational migrants, showed a negative effect with overall parasite intensity suggesting that the infections with haemosporidians lowers haematocrit. A recent field study of wild red-winged blackbirds population demonstrated that birds with higher *Plasmodium* and *Haemoproteus* parasitemia have lower hematocrit and a higher rate of red blood cell production [61]. The probability of *Plasmodium* infection showed a slight increase with Hct, probably as a result of upregulation of erythrocyte production, although, this warrants further investigation. *Plasmodium* remained absent in high elevation environments in resident birds and showed distinctly low prevalence in migrant populations. Given that all infections in our study are identified at the parasite genus level and we have not been able to detect differences in pathogenicity within a genus, the pathogenicity could be related to specific parasite lineages which could in turn have a variable effect [69]. In general, *Haemoproteus* has lower pathogenicity on its host than *Plasmodium* [70–72]. It is possible that *Plasmodium* infections are pathogenic to high elevation residents, which is reflected by low incidence at high elevations (but see below). Finally, some host species may be more susceptible to infection than others, and the species chosen for this study may have co-evolved with *Leucocytozoon* spp. parasites becoming resistant to them [73], a factor that probably explains the generally high prevalence of this parasite genus in western Himalayan birds. The high prevalence of *Leucocytozoon* spp. throughout the elevational gradient is in contrast with other studies (e.g. [74]) where *Leucocytozoon* showed an association with high elevation rather than in adjacent lowlands. In our western Himalayan system, there was no difference in infection status but intensity showed a sharp decline across the elevational gradient which points towards the role of high intensity infections lowering Hct and Hb levels at high elevations.

From an ecological perspective, climate change increases the risk of malaria in many birds [75]. In addition to rising mean temperatures, diurnal fluctuations in temperature have also been shown to affect the rate of parasite development [76] and range expansion, or allows a longer breeding season for arthropod vectors, both of which can alter transmission rates of malaria parasites. The relatively low prevalence of *Plasmodium* in high elevation environments could be attributed to micro-climatic differences governing the vector populations between low and high elevations and therefore influencing the spread of the disease across elevational gradients. The high elevation Himalayan bird community may not generally be exposed to vectors and parasites throughout the year—*Plasmodium* transmission is restricted by thermal constraints and distribution of potential vectors [28], and *Plasmodium* spp. was not found in the resident birds in the high elevation sites and was limited to the elevational migrants. With rising temperature predicted by imminent climate warming [77], and the subsequent range expansion of mosquito species and *Plasmodium* spp. parasites to high elevations, this might pose significant risks to naïve bird populations (e.g. Hawaii islands; [71]).

## Conclusions

Our study explored the links between seasonal migration, hypoxia physiology and disease dynamics. We found a higher parasite intensity in the non-breeding season regardless of migratory strategy, which is in line with previous studies suggesting that winter induces the relapse of the previous year’s infections. Our results show that high intensity in mixed haemosporidian infections have the predicted negative effect on oxygen-carrying capacity of blood after controlling for host species. Future work on chronic parasite infection must consider parasite intensity, rather than only relying on infection status. These results imply a role of elevational migration in disease dynamics. The potentially increased exposure to high intensity infections with upslope disease spread in the face of climate change will exacerbate hypoxic stress experienced by high elevation birds.

## Additional files

**Additional file 1: Table S1.** Summary of birds sampled, infection status, elevation and season.

**Additional file 2: Table S2.** Summary haematocrit values for birds sampled, **Figure S1.** Summary of Haemoglobin (Hb) concentration by infection status in Himalayan birds.

**Additional file 3: Table S3.** Ranking of candidate models.

## Abbreviations

a.s.l.: above sea level
GLMMs: generalized linear mixed models
Hb: haemoglobin
Hct: haematocrit
PCR: polymerase chain reaction
qPCR: quantitative PCR
RQ: relative quantification
