# Supplementary material for: Do avian blood parasites influence hypoxia physiology in a high elevation environment?
Source: BMC Ecol. 2018 May 14;18:15. doi: 10.1186/s12898-018-0171-2 (PMC5950187; doi:10.1186/s12898-018-0171-2)
Supplement: Supplementary file 2 — Additional file 2: Table S2. Summary haematocrit values for birds sampled, Figure S1. Summary of Haemoglobin (Hb) concentration by infection status in Himalayan birds. [file 12898_2018_171_MOESM2_ESM.docx]

Additional Information:

**Do avian blood parasites influence hypoxia physiology in a high elevation environment?**

Farah Ishtiaq^1*^ and Sahas Barve^2^

^1^Centre for Ecological Sciences, Indian Institute of Science, Bangalore 560012, India

^2^Department of Biological Sciences, Old Dominion University, Virginia, USA

Email: sahasbarve@gmail.com

***Corresponding author: Email:** [**ishtiaq.farah@gmail.com**](mailto:ishtiaq.farah@gmail.com)

Tel: +91 80 22932507

Fax: +91 80 23601428

Additional file S2. Summary haematocrit values for birds sampled

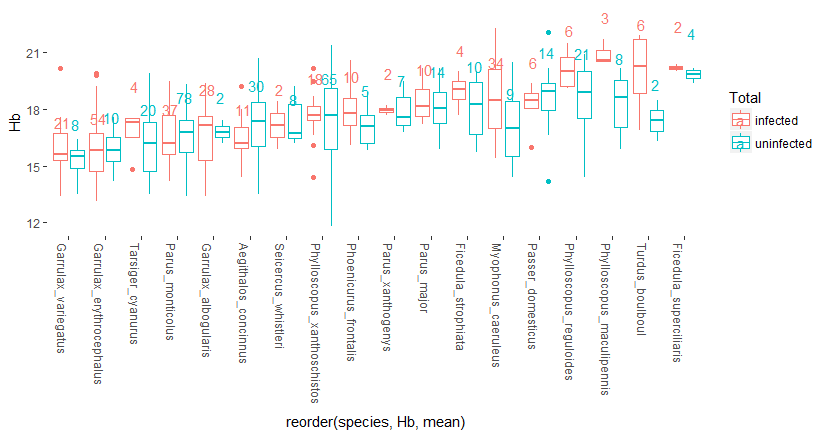


Fig. S1. Summary of Haemoglobin (Hb) concentration by infection status in Himalayan birds.
