# Supplementary material for: Do avian blood parasites influence hypoxia physiology in a high elevation environment?
Source: BMC Ecol. 2018 May 14;18:15. doi: 10.1186/s12898-018-0171-2 (PMC5950187; doi:10.1186/s12898-018-0171-2)
Supplement: Supplementary file 3 — Additional file 3: Table S3. Ranking of candidate models. [file 12898_2018_171_MOESM3_ESM.docx]

Additional Information:

**Do avian blood parasites influence hypoxia physiology in a high elevation environment?**

Farah Ishtiaq^1*^ and Sahas Barve^2^

^1^Centre for Ecological Sciences, Indian Institute of Science, Bangalore 560012, India

^2^Department of Biological Sciences, Old Dominion University, Virginia, USA

Email: sahasbarve@gmail.com

***Corresponding author: Email:** [**ishtiaq.farah@gmail.com**](mailto:ishtiaq.farah@gmail.com)

Tel: +91 80 22932507

Fax: +91 80 23601428

# Additional file S3 Ranking of candidate models (representing all possible combinations of the four predictors in each global model) by AICc, along with relative log-likelihood (RLL), ΔAICc, and model Akaike weights (*w_i_*) for:

# *Leucocytozoon* infected birds

| **Candidate model** | **d.f.** | **logLike** | **AICc** | **ΔAICc** | ***w_i_*** |
| --- | --- | --- | --- | --- | --- |
| 1. Hb ~ (L_intensity*breed)+elev+EM+ (1\|species) | 9 | −422.3 | 863.9 | 0.00 | 0.24 |
| 2. Hb ~ L_intensity+EM+breed+elev+ (1\|species) | 8 | −423.9 | 864.5 | 0.90 | 0.15 |
| 3. Hb ~ elev+ (1\|species) | 5 | −427.6 | 865.5 | 1.91 | 0.09 |
| 4. Hb ~ L_intensity*(EM+breed)+elev+(1\|species) | 10 | −422.3 | 865.7 | 2.13 | 0.08 |
| 5. Hb ~ L_intensity*(elev+breed)+ EM+(1\|species) | 11 | −421.4 | 866.2 | 2.59 | 0.06 |
| 6. Hb ~ L_intensity*elev+(1\|species) | 8 | −424.8 | 866.3 | 2.67 | 0.06 |
| 7. Hb ~ L_intensity*(breed+elev)+ (1\|species) | 10 | −422.7 | 866.5 | 2.86 | 0.05 |
| 8. Hb ~ (L_intensity*EM)+breed+elev+ (1\|species) | 9 | −423.8 | 866.6 | 2.99 | 0.05 |
| 9. Hb ~ (L_intensity*elev)+ breed+EM+(1\|species) | 10 | −423.3 | 867.8 | 4.14 | 0.03 |
| 10. Hb ~ L_intensity*breed+(1\|species) | 6 | −427.8 | 868 | 4.4 | 0.02 |
| 11. Hb ~ L_intensity*(EM+elev)+ (1\|species) | 10 | −423.5 | 868.1 | 4.53 | 0.02 |
| 12. Hb ~ L_intensity+ (1\|species) | 4 | −430.0 | 868.3 | 4.65 | 0.02 |
| 13. Hb ~ L_intensity*(EM+elev+breed)+ (1\|species) | 12 | −421.4 | 868.3 | 4.73 | 0.02 |
| 14. Hb ~ L_intensity*(EM+elev)+ breed+(1\|species) | 11 | −423.2 | 869.7 | 6.1 | 0.11 |
| 15. Hb ~ breed+ (1\|species) | 4 | −431.0 | 870.4 | 6.75 | 0.008 |
| 16. Hb ~ L_intensity*(EM+breed)+(1\|species) | 8 | −427.3 | 871.3 | 7.68 | 0.005 |
| 17. Hb ~ L_intensity*EM+ (1\|species) | 6 | −429.5 | 871.5 | 7.91 | 0.005 |
| 18. Hb ~ EM + (1\|species) | 4 | −433.6 | 875.5 | 11.85 | 0.001 |

L_intensity=*Leucocytozoon* intensity; EM= elevational migrant (1), resident (0); breed=Breeding season (1), non-breeding season (0)

# *Haemoproteus* infected birds

| **Candidate model** | **d.f.** | **logLike** | **AICc** | **ΔAICc** | ***w_i_*** |
| --- | --- | --- | --- | --- | --- |
| 1. Hb ~ EM+ (1\|species) | 6 | −109.0 | 231.8 | 0.00 | 0.23 |
| 2. Hb ~ H_intensity + (1\|species) | 4 | −111.6 | 232 | 0.27 | 0.20 |
| 3. Hb ~ breed+ (1\|species) | 4 | −112.1 | 233 | 1.23 | 0.12 |
| 4. Hb ~ EM+ (1\|species) | 4 | −112.2 | 233.3 | 1.57 | 0.10 |
| 5. Hb ~ H_intensity*breed + (1\|species) | 6 | −109.9 | 233.5 | 1.77 | 0.09 |
| 6. Hb ~ H_intensity*(breed+EM)+ (1\|species) | 8 | −107.3 | 233.7 | 1.91 | 0.08 |
| 7. Hb ~ elev+ (1\|species) | 5 | −111.5 | 234.2 | 2.46 | 0.06 |
| 8. Hb ~ H_intensity*(breed+EM)+elev+ (1\|species) | 10 | −106.0 | 236.6 | 4.85 | 0.02 |
| 9. Hb ~ (H_intensity*breed)+EM+elev+ (1\|species) | 9 | −107.4 | 236.6 | 4.89 | 0.02 |
| 10. Hb ~ (H_intensity*EM)+breed+ elev+ (1\|species) | 9 | −107.5 | 236.8 | 5.02 | 0.01 |
| 11. Hb ~ H_intensity +EM+breed+elev+ (1\|species) | 8 | −109.4 | 237.9 | 6.11 | 0.01 |
| 12. Hb ~ H_intensity*(EM+elev)+ (1\|species) | 10 | −107.1 | 238.9 | 7.13 | 0.007 |
| 13. Hb ~ H_intensity*elev+ (1\|species) | 8 | −110.0 | 239 | 7.28 | 0.006 |
| 14. Hb ~ H_intensity*(EM+elev)+breed+ (1\|species) | 11 | −107.0 | 241.7 | 9.96 | 0.002 |
| 15. Hb ~ H_intensity*(elev + breed) + (1\|species) | 10 | −108.7 | 242.0 | 10.27 | 0.001 |
| 16. Hb ~ (H_intensity*elev)+ EM+ breed+(1\|species) | 10 | −108.7 | 242.1 | 10.32 | 0.001 |
| 17. Hb ~ H_intensity *(breed+elev)+EM+ (1\|species) | 11 | −107.4 | 242.5 | 10.79 | 0.001 |
| 18. Hb ~ H_intensity*(elev+breed+EM)+ (1\|species) | 11 | −105.9 | 242.8 | 11 | 0.001 |

H_intensity=*Haemoproteus* intensity; EM= elevational migrant (1), resident (0); breed=Breeding season (1), non-breeding season (0)

# *Plasmodium* infected birds

| **Candidate model** | **d.f.** | **logLike** | **AICc** | **ΔAICc** | ***w_i_*** |
| --- | --- | --- | --- | --- | --- |
| 1. Hb ~EM+(1\|species) | 4 | −27.99 | 67.1 | 0.00 | 0.59 |
| 2. Hb ~ breed+ (1\|species) | 4 | −28.99 | 69.1 | 1.99 | 0.22 |
| 3. Hb ~ elev+(1\|species) | 5 | −28.45 | 71.9 | 4.84 | 0.05 |
| 4. Hb ~ P_intensity+(1\|species) | 4 | −30.47 | 72 | 4.95 | 0.05 |
| 5. Hb ~ P_intensity *breed+ (1\|species) | 6 | −26.34 | 72.3 | 5.25 | 0.04 |
| 6. Hb ~ P_intensity*EM+ (1\|species) | 6 | −26.56 | 72.8 | 5.69 | 0.03 |
| 7. Hb ~ P_intensity*(EM+breed) + (1\|species) | 8 | −24.37 | 80.7 | 13.68 | 0.01 |
| 8. Hb ~ P_intensity+EM+breed+elev+ (1\|species) | 8 | −24.67 | 81.3 | 14.27 | 0 |
| 9. Hb ~ (P_intensity*breed)+(elev+EM)+ (1\|species) | 9 | −22.32 | 85.1 | 18.07 | 0 |
| 10. Hb ~ P_intensity*elev+ (1\|species) | 8 | −27.62 | 87.2 | 20.17 | 0 |
| 11. Hb ~ (P_intensity*EM)+breed+ elev)+(1\|species) | 9 | −24.62 | 89.7 | 22.67 | 0 |
| 12. Hb ~ P_intensity*(EM+elev)+ (1\|species) | 10 | −19.88 | 91.2 | 24.12 | 0 |
| 13. Hb ~ (P_intensity*elev)+breed + EM+ (1\|species) | 10 | −20.32 | 92.1 | 25 | 0 |
| 14. Hb ~ P_intensity*(elev+breed)+ (1\|species) | 10 | −20.35 | 92.1 | 25.07 | 0 |
| 15. Hb ~ P_intensity*(breed + EM)+elev+ (1\|species) | 10 | −20.66 | 92.8 | 25.69 | 0 |
| 16. Hb ~ P_intensity*(EM+elev)+breed + (1\|species) | 11 | −19.94 | 105.6 | 38.82 | 0 |
| 17. Hb ~ P_intensity*(breed+elev)+ EM+ (1\|species) | 11 | −20.13 | 106.3 | 39.2 | 0 |
| 18. Hb ~ P_intensity*(elev+EM+breed)+ (1\|species) | 12 | −18.16 | 122.7 | 55.66 | 0 |

P_intensity=*Plasmodium* intensity; EM= elevational migrant (1), resident (0); breed=Breeding season (1), non-breeding season (0)

1. **Total infections**

| **Candidate model** | **d.f.** | **logLike** | **AICc** | **ΔAICc** | ***w_i_*** |
| --- | --- | --- | --- | --- | --- |
| 1. Hb ~(intensity*breed) +EM+elev+(1\|species) | 9 | −469.87 | 958.5 | 0.00 | 0.38 |
| 2. Hb ~ intensity*(breed+EM)+elev+ (1\|species) | 10 | −469.74 | 960.4 | 1.91 | 0.14 |
| 3. Hb ~ intensity+elev+EM+breed+(1\|species) | 8 | −472.08 | 960.7 | 2.27 | 0.12 |
| 4. Hb ~ elev+(1\|species) | 5 | −475.58 | 961.4 | 2.94 | 0.08 |
| 5. Hb ~ intensity *(breed+elev)+EM+ (1\|species) | 11 | −469.70 | 962.5 | 4.01 | 0.05 |
| 6. Hb ~ (intensity*EM)+ breed+elev+(1\|species) | 9 | −471.90 | 962.5 | 4.07 | 0.05 |
| 7. Hb ~ (intensity*elev)+EM+breed + (1\|species) | 10 | −471.42 | 963.7 | 5.27 | 0.02 |
| 8. Hb ~ intensity*(elev+breed)+ (1\|species) | 10 | −471.59 | 964.1 | 5.6 | 0.02 |
| 9. Hb ~ intensity*(breed+elev+EM)+ (1\|species) | 12 | −469.49 | 964.3 | 5.79 | 0.02 |
| 10. Hb ~ intensity*(EM+elev)+ (1\|species) | 10 | −471.95 | 964.8 | 6.33 | 0.01 |
| 11. Hb ~ intensity*(EM+breed)+(1\|species) | 8 | −474.21 | 965 | 6.54 | 0.01 |
| 12. Hb ~ intensity*(EM+elev)+breed+ (1\|species) | 11 | −471.08 | 965.2 | 6.77 | 0.01 |
| 13. Hb ~ intensity*elev+ (1\|species) | 8 | −474.37 | 965.3 | 6.85 | 0.01 |
| 14. Hb ~ breed+ (1\|species) | 4 | −479.48 | 967.1 | 8.65 | 0.00 |
| 15. Hb ~ intensity*breed + (1\|species) | 6 | −477.46 | 967.3 | 8.79 | 0.00 |
| 16. Hb ~ intensity*EM+ (1\|species) | 6 | −477.89 | 968.1 | 9.64 | 0.00 |
| 17. Hb ~ EM+ (1\|species) | 4 | −480.15 | 968.5 | 10 | 0.00 |
| 18. Hb ~ intensity+ (1\|species) | 4 | −481.60 | 971.4 | 12.89 | 0.00 |

1. **Mixed infections**

| **Candidate model** | **d.f.** | **logLike** | **AICc** | **ΔAICc** | ***w_i_*** |
| --- | --- | --- | --- | --- | --- |
| 1. Hb ~intensity+(1\|species) | 4 | −76.23 | 161.6 | 0.00 | 0.64 |
| 2. Hb ~ intensity*EM+ (1\|species) | 6 | −75.20 | 164.8 | 3.25 | 0.12 |
| 3. Hb ~ intensity*breed+(1\|species) | 6 | −75.91 | 166.2 | 4.69 | 0.02 |
| 4. Hb ~ elev+(1\|species) | 5 | −77.61 | 166.9 | 5.35 | 0.04 |
| 5. Hb ~ EM+ (1\|species) | 4 | −79.43 | 167.9 | 6.39 | 0.02 |
| 6. Hb ~ breed+(1\|species) | 4 | −79.49 | 168.1 | 6.52 | 0.02 |
| 7. Hb ~ intensity+elev+EM+breed + (1\|species) | 8 | −74.00 | 168.4 | 6.83 | 0.02 |
| 8. Hb ~ intensity*elev+ (1\|species) | 8 | −74.19 | 168.8 | 7.2 | 0.01 |
| 9. Hb ~ intensity*(breed+EM)+ (1\|species) | 8 | −74.53 | 169.4 | 7.88 | 0.01 |
| 10. Hb ~ (intensity*breed)+EM+elev+ (1\|species) | 9 | −73.31 | 170.3 | 8.7 | 0.00 |
| 11. Hb ~ (intensity*EM)+breed+elev+(1\|species) | 9 | −73.75 | 171.1 | 9.58 | 0.00 |
| 12. Hb ~ intensity*(elev+breed)+ (1\|species) | 10 | −72.54 | 172.2 | 10.64 | 0.00 |
| 13. Hb ~intensity*(breed+EM)+elev+ (1\|species) | 10 | −72.94 | 173 | 11.44 | 0.00 |
| 14. Hb ~ intensity*(EM+elev)+ (1\|species) | 10 | −73.33 | 173.8 | 12.21 | 0.00 |
| 15. Hb ~ intensity*(breed + elev) + EM+(1\|species) | 11 | −71.64 | 174.1 | 12.53 | 0.00 |
| 16. Hb ~ (intensity*elev)+breed+EM+ (1\|species) | 10 | −73.55 | 174.2 | 12.65 | 0.00 |
| 17. Hb ~ intensity+EM+breed+elev+ (1\|species) | 12 | −71.09 | 176.9 | 15.39 | 0.00 |
| 18. Hb ~ intensity*(EM+elev)+ breed+(1\|species) | 11 | −73.21 | 177.2 | 15.69 | 0.00 |
